# Self-Management and Clinical Decision Support for Patients With Complex Chronic Conditions Through the Use of Smartphone-Based Telemonitoring: Randomized Controlled Trial Protocol

**DOI:** 10.2196/resprot.8367

**Published:** 2017-11-21

**Authors:** Emily Seto, Patrick Ware, Alexander G Logan, Joseph A Cafazzo, Kenneth R Chapman, Phillip Segal, Heather J Ross

**Affiliations:** ^1^ Institute of Health Policy, Management and Evaluation University of Toronto Toronto, ON Canada; ^2^ Centre for Global eHealth Innovation Techna Institute University Health Network Toronto, ON Canada; ^3^ Lunenfeld-Tanenbaum Research Institute Mount Sinai Hospital Toronto, ON Canada; ^4^ Division of Nephrology Mount Sinai Hospital Toronto, ON Canada; ^5^ Division of Nephrology University Health Network Toronto, ON Canada; ^6^ Institute of Biomaterials and Biomedical Engineering University of Toronto Toronto, ON Canada; ^7^ Asthma and Airway Centre University Health Network Toronto, ON Canada; ^8^ Division of Endocrinology University Health Network Toronto, ON Canada; ^9^ Ted Rogers Centre of Excellence for Heart Function University Health Network Toronto, ON Canada

**Keywords:** mHealth, smartphone, multiple chronic diseases, randomized controlled trial

## Abstract

**Background:**

The rising prevalence of chronic illnesses hinders the sustainability of the health care system because of the high cost of frequent hospitalizations of patients with complex chronic conditions. Clinical trials have demonstrated that telemonitoring can improve health outcomes, but they have generally been limited to single conditions such as diabetes, hypertension, or heart failure. Few studies have examined the impact of telemonitoring on complex patients with multiple chronic conditions, although these patients may benefit the most from this technology.

**Objective:**

The aim of this study is to investigate the impact of a smartphone-based telemonitoring system on the clinical care and health outcomes of complex patients across several chronic conditions.

**Methods:**

A mixed-methods, 6-month randomized controlled trial (RCT) of a smartphone-based telemonitoring system is being conducted in specialty clinics. The study will include patients who have been diagnosed with one or more of any of the following conditions: heart failure, chronic obstructive pulmonary disease, chronic kidney disease, uncontrolled hypertension, or insulin-requiring diabetes. The primary outcome will be the health status of patients as measured with SF-36. Patients will be randomly assigned to either the control group receiving usual care (n=73) or the group using the smartphone-based telemonitoring system in addition to usual care (n=73).

**Results:**

Participants are currently being recruited for the trial. Data collection is anticipated to be completed by the fall of 2018.

**Conclusions:**

This RCT will be among the first trials to provide evidence of the impact of telemonitoring on costs and health outcomes of complex patients who may have multiple chronic conditions.

**Trial Registration:**

International Standard Randomized Controlled Trial Number (ISRCTN): 41238563; http://www.isrctn.com/ISRCTN41238563 (Archived by WebCite at http://www.webcitation.org/6ug2Sk0af) and Clinicaltrials.gov NCT03127852; https://clinicaltrials.gov/ct2/show/NCT03127852 (Archived by WebCite at http://www.webcitation.org/6uvjNosBC)

## Introduction

Patients with chronic illnesses, particularly those with multiple chronic conditions (MCCs), face numerous challenges in the self-management of their conditions, including complex decision making, varying and often conflicting clinical management advice, and frequent hospitalizations. A 2011 Canadian Institute for Health Information study found that 3 out of 4 Canadians aged 65 years and older reported having at least one chronic condition, whereas 1 in 4 seniors reported having 3 or more conditions [1]. Although 75% of all health care costs are solely devoted to managing chronic illnesses, 5% of the population who are patients with complex conditions consumes more than 50% of all dollars devoted to health care [2]. With the increasing prevalence of chronic illnesses and MCCs [1], the sustainability of our health care system is threatened. However, through enhanced patient self-care and clinical management, considerable reductions in health care spending and improved health outcomes could be achieved.

Digital health tools may empower patients and their informal caregivers (ie, family and friends) in more effective self-management of MCCs and serve as a critical decision support tool for their health care providers (HCPs). In particular, telemonitoring enables patients to track their vital signs and symptoms and to receive automated self-care instructions. The automated instructions can be based on current physiological measurements, self-monitored symptoms, and readily analyzed trends in both [3]. Automated real-time alerts and frequently collected and analyzed physiological data can also support clinical decisions by HCPs [3].

A growing body of research on telemonitoring interventions exists, but it focuses on single conditions such as diabetes, hypertension, or heart failure (HF) [4-6]. Several studies and systematic reviews have shown that the use of telemonitoring interventions for the management of chronic conditions leads to positive health outcomes and significant reductions in health care costs [7-11]. Contradictory studies that did not find improvements often had interventions that excluded a self-care component, were difficult to use, were not adhered to by patients, or did not target the most frequently hospitalized patients [12-16]. Due to the added complexity involved, few studies have investigated the application of telemonitoring to high-risk patients with MCCs, although these patients may benefit the most from such an intervention [17-22].

This randomized controlled trial (RCT) will investigate the use of a smartphone-based telemonitoring system for the management of complex patients. Complex patients will be defined as those who are at high risk for hospitalization, exacerbations of their chronic conditions, and disease progression, as well as patients with MCCs. Specifically, patients with HF, chronic obstructive pulmonary disease (COPD), uncontrolled hypertension, chronic kidney disease (CKD), insulin-dependent diabetes, and combinations of these chronic conditions will be included in the study because of the high prevalence and costs associated with these conditions. The central research question is as follows: what is the impact of a smartphone-based telemonitoring system for patients with complex chronic illnesses on health status (with SF-36 as the primary outcome measure)? Secondary outcome measures will include cost, self-management, clinical management, health outcomes, and health service utilization.

## Methods

### Research Ethics Board Approval

This study has received approval from the Research Ethics Board at the University Health Network (15-9995-BE) and Mount Sinai Hospital (MSH REB 16-0093-E).

### Patient Inclusion and Exclusion Criteria

The patient inclusion and exclusion criteria are provided in Textboxes 1 and 2, respectively.

### Patient Recruitment

Patients will be recruited during regularly scheduled visits to the University Health Network or Mount Sinai Hospital clinics associated with their chronic illnesses. After the clinician determines that the patient is eligible for recruitment, the clinician will ask the patient if they are willing to speak to the study coordinator regarding the study. All eligible participants based on the inclusion and exclusion criteria will be asked to sign a written consent form before being enrolled in the study. After the randomization into the intervention or control groups, the study coordinator will provide the patients in the intervention group and, if appropriate, their caregivers with training on the telemonitoring system. Patients will be compensated Can $24 for their participation in the study, which will cover the cost of parking for one clinic visit.

**Inclusion criteria of patients for the study.**

- Adults (aged 18 years or older)
- Diagnosed with one or more of the following and with the indicated criteria:
  - Heart failure (HF)
    - Followed by a cardiologist at the Ted Rogers Centre of Excellence for Heart Function, University Health Network, who has the primary responsibility for management of the patient’s HF
    - Reduced ejection fraction (EF<0.40)
  - Chronic obstructive pulmonary disease (COPD)
    - Followed by a respirologist at the Asthma and Airway Centre, University Health Network, who has primary responsibility for management of the patient’s COPD
    - Spirometrically confirmed diagnosis of COPD of Global Initiative for Chronic Obstructive Lung Disease (GOLD) stage II or higher (defined as postbronchodilator forced expiratory volume in 1 s [FEV_1_] <80% predicted and FEV_1_/FVC ratio <70%, where FVC is the forced vital capacity)
    - Smoking history of >=20 pack-years or homozygous alpha-1 antitrypsin deficiency
    - Prescribed an action plan for the early self-treatment of acute exacerbations
  - Uncontrolled hypertension
    - Followed by a hypertension specialist at the Mount Sinai Hospital, who has primary responsibility for management of the patient’s hypertension
    - For patients without diabetes: blood pressure ≥140/90 mmHg auscultatory (manual measurement) or ≥135/85 mmHg oscillometric (automated measurement)
    - For patients with diabetes: blood pressure ≥130/80 mmHg
    - On two or more blood pressure lowering medications
  - Diabetes
    - Followed by an endocrinologist at the University Health Network, who has primary responsibility for management of the patient’s diabetes
    - Insulin-requiring diabetes (Type 1 or Type 2)
    - Performing self-capillary glucose monitoring
  - Chronic kidney disease (CKD)
    - Followed by a hypertension specialist at the Mount Sinai Hospital, who has primary responsibility for management of the patient’s CKD
    - Grade 3b-5 (estimated glomerular filtration rate<45 mL/1.73 m^2^)
    - Must have uncontrolled hypertension
- Patient or their caregiver speaks and reads English adequately to provide informed consent and understand the text prompts in the smartphone app
- Ability to comply with instructions using the telemonitoring system (eg, able to stand on the weighing scale and able to answer symptom questions)

**Exclusion criteria of patients for the study.**

Exclusion criteria

- Patients on mechanical circulatory support
- Patients on the heart transplant list
- Terminal diagnosis with life expectancy <1 year
- Dementia or uncontrolled psychiatric illness
- Resident of a long-term care facility
- On dialysis
- Unable to provide informed consent
- Unable to speak or read English

To perform the randomization, the Web-based computer-generated randomization tool, Research Randomizer (Social Psychology Network, USA, [23]) will be used to generate the sequence of intervention and control group allocation. The study coordinator performing the recruitment will be blinded until the patient has consented to participate. After obtaining consent, the study coordinator will take the top card of the prepared stack of randomization cards and will tell the patient if they are in the intervention or control group. The participants will be block randomized (blocks of 4) and stratified into groups according to their primary condition (HF, COPD, hypertension, and diabetes). The HF group will further be stratified on the basis of New York Heart Association (NYHA) classification (NYHA class 2-3, NYHA class 4), the hypertensive patients will be stratified to those with and without diabetes, and the hypertensive patients will also be stratified to those with and without CKD. The stratification of the hypertensive patients is performed because the coordination of clinical management for the combination of hypertension and CKD and of hypertension and diabetes was established between specialists who were willing to participate in this study.

In addition to asking patients whether they are interested in participating in the study during their regularly scheduled clinic visit, an additional recruitment strategy may be employed to speed up patient enrolment. The research study coordinator, an independent third party who is not part of the clinical care team, will generate an eligible patient mail-out list. This list will be derived from the patient roster at the clinics. The list will be screened and verified by the clinic nurses/physicians to ensure appropriateness as determined by the study inclusion criteria. The research coordinator will mail-out an invitation letter including a study description and consent form. The study coordinator will arrange a time to discuss in person or over the phone with patients interested in learning more about the study.

### Sample Size Calculation

The primary outcome measure SF-36 was used to conduct a sample size calculation. Assuming a moderate effect size on the general health dimension of SF-36 of 10.5 points [24], a score of 57±21 for the control group [25], 80% power, and Cronbach alpha=.05 (two-sided), the sample size per group was calculated to be 63. Assuming that 15% of patients will be lost to follow-up (including mortality) [26,27], the sample size per group is 73. Hence, 146 patients will be enrolled during the trial and randomized 1:1 into control and telemonitoring groups.

### Telemonitoring System

**Figure 1:**
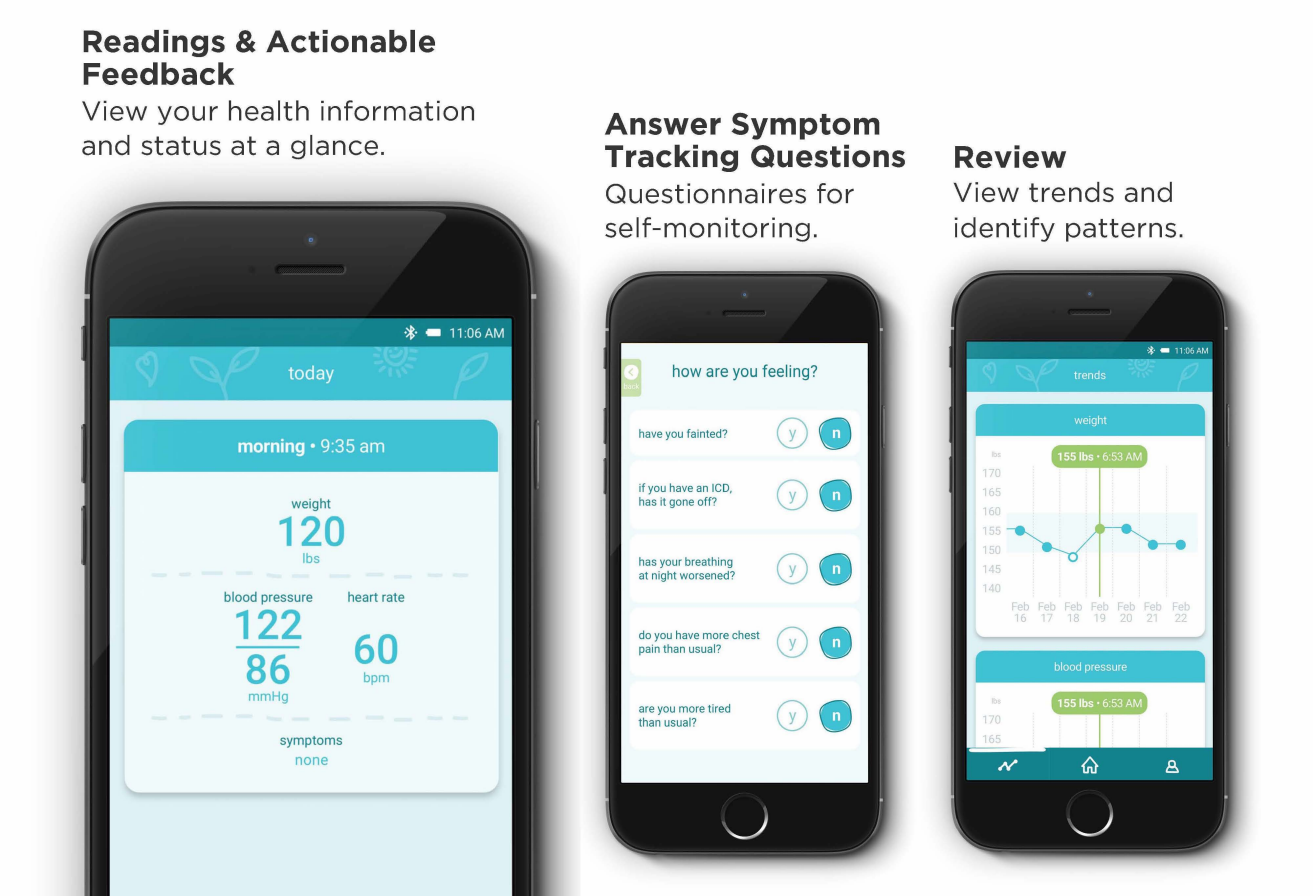
Medly app screenshots.

The telemonitoring system, named *Medly*, will enable patients with complex chronic illnesses, including MCCs, to take clinically relevant physiological measurements with wireless home medical devices and to answer symptom questions on the smartphone (Figure 1). The measurements will be automatically and wirelessly transmitted to the smartphone and then to a data server. Specifically, patients with HF will monitor daily weight (A&D Medical Bluetooth weighing scales), blood pressure/heart rate (A&D Medical Bluetooth blood pressure monitors), and symptoms; patients with COPD will provide self-reported symptoms; hypertensive patients will monitor their blood pressure; patients with diabetes will monitor blood sugar levels (iBGStar Bluetooth blood glucose meters); and patients with CKD will monitor blood pressure. Automated self-care instructions or messages that have been carefully developed with health care specialists will be sent to the patient based on the readings and reported symptoms [28]. If there are signs of deteriorating health of a patient, an alert will be sent to a clinician responsible for the particular chronic condition of concern in the respective specialty clinic(s) as appropriate. All the relevant patient data will be sent to the clinicians via an email alert, and they will be able to access historical and trending data of their patients through a secure Web portal.

### Study Design

The control group will receive usual care, and the intervention group will receive usual care plus the telemonitoring intervention. Both groups will be followed for 6 months. Each intervention study participant will be provided with a smartphone. This phone will be loaded with the app and a limited data plan to enable data transfer. Patients will also be provided with relevant home medical monitoring devices (eg, weighing scales and blood pressure monitors for patients with HF, and blood pressure monitors for hypertensive patients). Patients will be asked to take measurements and record symptoms at a specified frequency at their homes. Home measurement frequency depends on the specific chronic condition(s). Patients with HF and COPD are generally asked to take daily measurements, whereas patients with hypertension and CKD take blood pressure readings several times per week as specified by the algorithm developed in conjunction with specialists. Patients with diabetes will be asked to follow the frequency of blood glucose measurements as recommended by their endocrinologist. Patients will be notified when they should take measurements at home through the smartphone app. Patient-specific baseline information will be entered into the telemonitoring system dashboard, such as an individual target weight range, target blood pressure range, medication list, and COPD action plan, before the patient starts using *Medly* at home.

### Outcome Measures

The primary outcome will be health status as measured with SF-36 because this metric will be relevant to all chronic conditions under investigation. A secondary outcome measure will be the cost (from the health system and patient perspectives). The cost of health care will include hospitalizations, emergency department (ED) visits, clinic visits, medications, and the telemonitoring program as relevant to the different chronic illnesses. Disease-specific subanalyses on cost will also be conducted.

Other secondary outcome measures will include combined hospitalization for any reason or death from any cause within 6 months after enrolment, hospitalization, mortality, days in hospital, number of ED and clinic visits, and medications. Secondary outcome measures will also include general quality of life as measured by the EuroQol 5D-RL, anxiety and depression as measured with the Hospital Anxiety and Depression Scale [29], and patients’ self-efficacy to self-manage their disease as measured with the Self-Efficacy for Managing Chronic Disease Scale [30]. See Table 1 for the assessment schedule.

The disease-specific outcome measures (generally obtained as standard of care) are listed below:

- Heart failure (HF)
  - Left ventricular ejection fraction
  - Brain natriuretic peptide
  - Self-care as measured by the Self-Care of Heart Failure Index [31]
  - HF-specific quality of life as measured by Minnesota Living with Heart Failure Questionnaire [32]
  - Shortness of breath as measured using a visual analogue scale for dyspnea
  - Blood work: creatinine, sodium, potassium, hemoglobin, and urate
  - Prognostic score as determined by the Seattle Health Failure Model (requires: age, gender, NYHA class, weight, ejection fraction, systolic blood pressure, medication list [including diuretics], lab results [hemoglobin, lymphocytes, uric acid, total cholesterol, and sodium], and QRS interval)
- Chronic obstructive pulmonary disease (COPD)
  - Pre- and postbronchodilator-forced expiratory volume in 1 s
  - Symptoms score from the telemonitoring system
  - COPD Assessment Test score [33]
  - COPD-specific knowledge after 6 months of app usage as measured by the Bristol COPD Knowledge Questionnaire [34]
  - Patient self-efficacy at 6 months as measured by the COPD Self-Efficacy Scale [35]
  - COPD severity as measured by the BODE Index [36] at 6 months
  - Annualized exacerbation rate categorized as mild (managed in the ambulatory setting), moderate (requiring emergency room care), or severe (requiring hospital care)
- Chronic kidney disease (CKD)
  - Estimated glomerular filtration rate
  - Symptoms score from the telemonitoring system
  - Blood pressure as measured by an automatic blood pressure monitor
- Hypertension
  - Blood pressure as measured by the blood pressure monitor at the clinic using an automated device such as BpTRU and at home with the home blood pressure monitor provided. The latter involves patients measuring their blood pressure at home using a validated Bluetooth-enabled home blood pressure device twice in the morning and twice in the evening daily for 7 consecutive days. The second reading values will be averaged and the mean of the 14 readings will be used to compare baseline and exit values to assess mean change.
- Diabetes
  - Hemoglobin A_1c_
  - Frequency and severity of hypoglycemia

### Data Acquisition

Number of hospitalizations, days in hospital, number of ED and clinic visits, and medications will be determined through the hospital electronic medical records and a manual chart review of all participants’ clinical records. Number of hospitalizations, days in hospital, and ED visits will be verified by patient participants through self-report with the help of a questionnaire. The cost of the intervention will be tracked, including equipment costs and human resources for clinical support, technical support, and program management. Standard cost values for a day in hospital, ED visit, etc will be used to estimate net cost savings or expenditures. Values for pre- and posttrial health outcome measures (eg, blood test results) will be obtained through hospital clinical records or through the *Medly* database for symptoms. Pre- and follow-up questionnaires (baseline, 1-month, and 6-months) will be administered to both control and intervention groups that contain the validated survey tools listed above. Participants will be able to complete the questionnaires at the clinic or complete it at home. Participants who choose to complete the questionnaire at home will be given a prepaid self-addressed envelope to mail the questionnaire back to the study team. The 1-month questionnaire will be given to participants upon enrolment with instructions to complete the questionnaire 1 month after starting the trial and to mail it back to the study team using the prepaid envelope. The study coordinator will call each participant in the 1-month period to remind participants to complete the questionnaire. Usage data, in terms of adherence to taking measurements and use of the different features, will also be data mined from the servers.

In addition to the quantitative metrics, all participants will be asked basic questions such as their self-care practice and their thoughts on telemonitoring at the start of the study. A subgroup of the intervention arm will also be interviewed individually to assess their experiences and perceptions regarding the use of *Medly* at the end of the study. The number of patients that will be interviewed will depend on when saturation of information is reached, typically 15-20 participants. The interviews will be conducted in a quiet and private space within a clinic (eg, consultation room or education room). Before the interview, participants will be informed that notes will be taken and they will be audiotaped for data analysis. The clinicians involved in the trial will also be asked to participate in interviews to determine their perceptions of *Medly*.

### Data Analysis

For the primary analyses, posttrial data and change scores will be compared between the control and intervention groups using independent Student *t* test and Mann–Whitney test (for normally and not normally distributed data, respectively) based on intention-to-treat. Paired Student *t* test and Wilcoxon signed rank test will be performed to compare baseline and poststudy data within the control and telemonitoring groups. Secondary analyses seeking to assess the impact of the telemonitoring system over time by incorporating the 1-month time point will be analyzed using general linear mixed model procedures. Randomization of study participants should yield an equal distribution of possible confounding variables. However, adjustments may be required for unexpected variations in the baseline characteristics between groups (eg, age and gender). Similarly, depending on the combinations of diseases of the participants, subgroup analyses may be performed. All statistical analyses will be performed using the statistical software app SPSS (IBM Corporation, USA).

The cost evaluation will be performed by comparing any savings through reductions in hospitalization, emergency, and clinic visits through the use of the intervention. This will further include amortizing the equipment costs and factoring in additional clinical, technical, and management resources. An analysis of indirect costs will also be performed, including such factors such as days absent from work.

Interview data will be analyzed using a conventional content analysis approach [37]. Two researchers will analyze the transcripts independently and will discuss the themes until a consensus is reached. The software program NVivo will be used to help organize the themes (QSR International, Doncaster, Victoria, Australia).

**Table 1:** Schedule of outcome assessments. X represents that data is collected for the specified outcome measure at that time point.

Outcome measures		Baseline	1 month	6 months
**Questionnaires**				
	SF-36 (primary outcome)	X	—	X
	Demographics	X	—	—
	EuroQol 5D-5L	X	X	X
	Hospital Anxiety and Depression Scale	X	—	X
	Self-Efficacy for Managing Chronic Disease Scale	X	—	X
	Self-Care of Heart Failure Index (only for patients with HF^a^)	X	X	X
	Minnesota Living with Heart Failure Questionnaire (only for patients with HF)	X	X	X
	Visual analogue scale (only for patients with HF)	X	X	X
	COPD^b^ Assessment Test (only for patients with COPD)	X	X	X
	Bristol COPD Knowledge Questionnaire (only for patients with COPD)	X	X	X
	BODE Index (COPD only)	X	X	X
**Health service utilization**				
	Number of hospitalizations in the previous 6 months	X	—	X
	Number of days in hospital in the previous 6 months	X	—	X
	Number of ED^c^ visits in the previous 6 months	X	—	X
	Number of clinic visits in the previous 6 months	X	—	X
**HF-specific clinical outcomes**				
	Left ventricular ejection fraction	X	—	X
	Brain natriuretic peptide	X	—	X
	Blood work: creatinine, sodium, potassium, hemoglobin, urate	X	—	X
	Seattle Health Failure Model	X	—	X
**COPD-specific clinical outcomes**				
	Pre- and postbronchodilator (FEV_1_)^d^	X	—	X
	Exacerbation rate in the previous 6 months	X	—	X
**CKD^e^-specific clinical outcomes**				
	Estimated glomerular filtration rate	X	—	X
	Blood pressure	X	—	X
**Hypertension-specific clinical outcomes**				
	Blood pressure	X	—	X
**Diabetes-specific clinical outcomes**				
	Hemoglobin A_1c_	X	—	X
	Frequency and severity of hypoglycemia in the previous 6 months	X	—	X

^a^HF: heart failure.

^b^COPD: chronic obstructive pulmonary disease.

^c^ED: emergency department.

^d^FEV_1_: forced expiratory volume in 1 s.

^e^CKD: chronic kidney disease.

## Results

Participants are currently being recruited for the trial. Due to longer than anticipated development of the *Medly* platform, the telemonitoring system could not be rolled out to patients across various chronic diseases as originally intended. *Medly* has been rolled out for patients with HF, and the functionality for telemonitoring of patients with hypertension is anticipated to be available by the end of 2017. Functionality for telemonitoring of the remaining chronic diseases will follow. As of November 1, 2017, a total of 60 patients with HF have been recruited into the trial. Data collection is anticipated to be completed by the end of 2018.

## Discussion

### Principal Findings

This RCT aims to investigate the impact of a smartphone-based telemonitoring system on patients with complex chronic conditions, including those with MCCs. The mixed methods design of the trial will enable triangulation from a variety of data sources, including questionnaires, chart reviews, and interviews.

Evidence from previous trials indicate that positive health outcomes are achievable from telemonitoring, particularly for patients who have the most severe conditions. However, very few clinical trials have been conducted to investigate the impact of telemonitoring across MCCs [17-22]. This is largely because of the following three reasons:

First, it is difficult to develop a telemonitoring platform that can be tailored to effectively manage several combinations of chronic conditions because of the disparate needs of different chronic conditions. We have used our experience of over 10 years of designing, developing, and evaluating apps for chronic disease management using a user-centred design process to develop the *Medly* platform.

Second, determining appropriate outcome measures that are appropriate across MCCs is difficult because many metrics are disease specific. For example, reduction in hospitalization may be the primary goal of telemonitoring of patients with HF but would not be a suitable measure for chronic diseases such as hypertension. Therefore, health status as measured by SF-36 was chosen as the primary outcome measure because it is relevant to all interested chronic conditions.

Third, performing a trial of patients with MCCs requires coordination of several specialities that are typically not integrated and do not have ideal communication channels between them. For our clinical trial, we have clinical specialists as part of the research team who will be managing the patient participants. These clinical specialists are very familiar with the telemonitoring system and have already worked together in the past to coordinate care for certain complex patients.

### Significance of Research

Patients with multiple chronic illnesses account for the largest expenditure of health care dollars and require the most support to manage their complex conditions. This RCT will be among the first to provide evidence of the impact of telemonitoring on costs and health outcomes of complex patients who may have MCCs. We anticipate that our health app for the management of MCCs will be a cost-effective and scalable tool that will improve health outcomes and quality of life, while empowering and reassuring patients and their informal caregivers.

## Abbreviations

CKD: chronic kidney disease
COPD: chronic obstructive pulmonary disease
ED: emergency department
FEV _1_: forced expiratory volume in 1 s
HCP: health care provider
HF: heart failure
MCCs: multiple chronic conditions
RCT: randomized controlled trial

## Appendix

Multimedia Appendix 1 CIHR peer-review comments.
