# Supplementary material for: Self-Management and Clinical Decision Support for Patients With Complex Chronic Conditions Through the Use of Smartphone-Based Telemonitoring: Randomized Controlled Trial Protocol
Source: JMIR Res Protoc. 2017 Nov 21;6(11):e229. doi: 10.2196/resprot.8367 (PMC5717446; doi:10.2196/resprot.8367)
Supplement: Multimedia Appendix 1 [file resprot_v6i11e229_app1.pdf]

February/Février 21, 2014

DC0190GP

Canadian Institutes of Health Research / Instituts de recherche en santé du Canada

Notice of Decision / Avis de décision

Application Number/Numéro de la demande: 316619
Committee Code/Code du comité: EHI

Applicants/Candidats: Dr. Emily SETO; Ms. Stephanie W ONG

With/Avec: Dr. J. CAFAZZO; Dr. D. CASEY; Prof. K. CHAPMAN; Dr. A. LOGAN; Dr. J. MILLER; Dr. P. RITVO; Dr. H. ROSS

Institution paid/ Établissement payé: University Health Network (Toronto)

Title/Titre: Self-management and clinical decision support of multiple chronic conditions through the use of mobile health telemonitoring

Primary Inst./ Inst. principal: Health Services and Policy Research

Other Related Inst./ Autres inst. connexes: Population and Public Health

**Competition Outcome/Résultats du concours:**	Catalyst Grant: eHealth Innovations<br>October/Octobre 15, 2013
**Number in competition/Nbre de demandes dans le concours:**	88
**Number approved/Nbre de demandes approuvées:**	12

**Decision on your application/ Décision sur votre demande:**	Approved
**Average annual amount/ Montant annuel moyen:**	$91,916
**Equipment amount/ Montant pour les appareils:**	$0
**Term/Durée:**	1 **yrs/ans** 0 **months/mois**

Peer Review Committee Recommendation, for your information and use/
Recommandation du comité d'examen par les pairs, pour fins d'information et d'utilisation:

**Committee/Comité:**	e-Health Innovations
**Application rank within the competition/ Rang de la demande dans ce concours:**	6
**Percent Rank Within the Competition/ Rang en pourcentage au sein du concours:**	6.82%
**Rating/ Cote:**	4.08
**Recommended average annual amount/ Montant annuel moyen recommandé:**	$91,916
**Recommended equipment amount/ Montant recommandé pour les appareils:**	$0

*** Applications receiving a score of less than 3.5 on any evaluation criteria will not be considered for Funding. / Les demandes qui ont reçu une note inférieure à 3.5 pour n'importe quel des critères d'évaluation ne sont pas admissibles.

This document is for information only. Official payment is stated on the CIHR Authorization for Funding.
Document à titre d'information seulement. Le paiement officiel est indiqué sur l'autorisation de financement des IRSC.

CIHR IRSC

Institute of Aboriginal Peoples' Health
Institute of Aging
Institute of Cancer Research
Institute of Circulatory and Respiratory Health
Institute of Gender and Health
Institute of Genetics
Institute of Health Services and Policy Research
Institute of Human Development and Child and Youth Health
Institute of Infection and Immunity
Institute of Musculoskeletal Health and Arthritis
Institute of Neurosciences, Mental Health and Addiction
Institute of Nutrition, Metabolism and Diabetes
Institute of Population and Public Health

Institut de la santé des Autochtones
Institut du vieillissement
Institut du cancer
Institut de la santé circulatoire et respiratoire
Institut de la santé des femmes et des hommes
Institut de génétique
Institut des services et des politiques de la santé
Institut du développement et de la santé des enfants et des adolescents
Institut des maladies infectieuses et immunitaires
Institut de l'appareil locomoteur et de l'arthrite
Institut des neurosciences, de la santé mentale et des toxicomanies
Institut de la nutrition, du métabolisme et du diabète
Institut de la santé publique et des populations

March 15, 2014

Dr. Emily SETO
190 Elizabeth Street
R Fraser Elliott Building, 4th Floor
Centre for Global eHealth Innovation
University Health Network
Toronto, Ontario M5G 2C4

Dear Dr. SETO:

We are pleased to inform you that the Canadian Institutes of Health Research (CIHR) has approved your recent application entitled "Self-management and clinical decision support of multiple chronic conditions through the use of mobile health telemonitoring". Since you are receiving this letter through ResearchNet your Authorization for Funding will follow in the mail. If you cannot access the review documents related to your proposal through ResearchNet, please contact the CIHR staff member listed below.

Please note that in addition to any budget cuts made by the peer review committee, an additional across-the-board administrative cut of approximately 7.76% was applied to the approved applications to maximize the number of applications funded.

Should you have any questions about the review process, please address them directly to CIHR staff. Do not contact the officers or members of the peer review committee. As CIHR does not notify co-applicants of the decision, we ask that you inform those individuals involved, along with their research institutions (if different from your own), of the outcome of this application.

If you have any questions, please contact Jeff Warren, Program Delivery Coordinator, at 613-948-2813 or jeff.warren@cihr-irsc.gc.ca.

Congratulations on your success in this competition!

Mary Fraser Valiquette

Mary Fraser Valiquette
A/Deputy Director, Program Delivery
Strategic Program Design and Analytics
Research and Knowledge Translation Portfolio

360142-201310CEH-EHI-316619-I006-113251-NCAA2

CIHR IRSC

Institute of Aboriginal Peoples' Health

Institute of Aging

Institute of Cancer Research

Institute of Circulatory and Respiratory Health

Institute of Gender and Health

Institute of Genetics

Institute of Health Services and Policy Research

Institute of Human Development and Child and Youth Health

Institute of Infection and Immunity

Institute of Musculoskeletal Health and Arthritis

Institute of Neurosciences, Mental Health and Addiction

Institute of Nutrition, Metabolism and Diabetes

Institute of Population and Public Health

Institut de la santé des Autochtones

Institut du vieillissement

Institut du cancer

Institut de la santé circulatoire et respiratoire

Institut de la santé des femmes et des hommes

Institut de génétique

Institut des services et des politiques de la santé

Institut du développement et de la santé des enfants et des adolescents

Institut des maladies infectieuses et immunitaires

Institut de l'appareil locomoteur et de l'arthrite

Institut des neurosciences, de la santé mentale et des toxicomanies

Institut de la nutrition, du métabolisme et du diabète

Institut de la santé publique et des populations

March 15, 2014

Dr. Emily SETO
190 Elizabeth Street
R Fraser Elliott Building, 4th Floor
Centre for Global eHealth Innovation
University Health Network
Toronto, Ontario M5G 2C4

Dear Dr. SETO:

Congratulations on your success in the recent Canadian Institutes of Health Research funding competition.

You should take great pride in this achievement, particularly given the highly competitive nature of CIHR peer review. Excellence in research will always rest on the shoulders of individual inspiration, curiosity, and drive. Given the Canadian health research community’s global reputation for excellence, I am confident that your work will help create new scientific knowledge that will strengthen the health care system for the benefit of Canadians.

As you know, peer review is the cornerstone of our research funding system. This process is made possible because of the kind volunteerism of individuals who generously gave their time to review your application. As a CIHR-funded researcher, you may be invited to serve in the peer review process as we are continuously recruiting and retaining the most accomplished, innovative and creative scientists to review health research proposals.

To meet CIHR goals, we must also continue to communicate the value of health research to Canadians. That is why we encourage you to work with your institution to promote your research. We have developed guidelines on public communication, available at: www.cihr-irsc.gc.ca/e/30789.html, to support you in this activity.

Once again, I offer you my congratulations and best wishes for success in your research.

Yours sincerely,

Alain Beaudet, MD, Ph.D.
President

359234-201310CEH-EHI-316619-I006-113251-CONGR

President

Canadian Institutes of Health Research
Room 97, 160 Elgin Steet, Address locator: 4809A
Ottawa, (Ontario) K1A 0W9 Tel.: (613) 941-2672
Fax (613) 954-1800 **www.cihr-irsc.gc.ca**

Président

Instituts de recherche en santé du Canada
Pièce 97, 160 rue Elgin, Indice de l'adresse: 4809A
Ottawa, (Ontario) K1A 0W9 Tél.: (613) 941-2672
Fax (613) 954-1800 **www.irsc-cihr.gc.ca**

Canada

Review Type/Type d'évaluation: Committee Member 1/Membre de comité 1
Name of Applicant/Nom du chercheur: SETO, Emily
Application No./Numéro de demande: 316619
Agency/Agence: CIHR/IRSC
Competition/Concours: 2013-10-15 Catalyst Grant: e-Health Innovations: Supporting More Efficient Population and Individualized Healthcare/Subvention catalyseur : Innovations en cybersanté : soutenir l'amélioration des soins de santé axés sur les individus et les populations
Committee/Comité: e-Health Innovations/Innovations en cybersanté
Title/Titre: Self-management and clinical decision support of multiple chronic conditions through the use of mobile health telemonitoring

---

Assessment/Évaluation:

Review of: SETO

Score: 3.6

1. **A brief synopsis of the proposal** *Medly* will enable patients with MCCs to take physiological measurements with wireless home medical devices and to answer symptom questions on the mobile phone. The measurements will be automatically and wirelessly transmitted to the mobile phone and then to a data server. Specifically, patients with HF will monitor daily weight and blood pressure/heart rate, CKD patients will monitor blood pressure, and all patients (including COPD) will monitor symptoms. A small feasibility pilot will be conducted to validate the tool, revealing any issues in clinical workflow, telemonitoring system design, methods for patient recruitment, etc. prior to the proposed randomized controlled trial (RCT). The telemonitoring platform named "*Medly*", will be specifically evaluated for three chronic conditions: HF, COPD, and CKD. HF and COPD are among the most common causes for avoidable hospitalizations (31), and CKD patients incur very high costs when hemodialysis is required (32, 33).

o the hypothesis to be tested, or the questions to be answered;

What is the impact of a telemonitoring system for patients with MCCs, on self-management, clinical outcomes and health service utilization? The study will include high-risk patients who account for the largest healthcare spending (i.e., high-risk for re-hospitalization; defined as having been hospitalized within the last 6 months).

o the objectives to be achieved and approach proposed;

A RCT will be conducted to determine the impact of *Medly* on healthcare costs and clinical

outcomes. The trial will be conducted at the HF, COPD, and CKD clinics at the University

Review Type/Type d'évaluation: Committee Member 1/Membre de comité 1
Name of Applicant/Nom du chercheur: SETO, Emily
Application No./Numéro de demande: 316619
Agency/Agence: CIHR/IRSC
Competition/Concours: 2013-10-15 Catalyst Grant: e-Health Innovations: Supporting More Efficient Population and Individualized Healthcare/Subvention catalyseur : Innovations en cybersanté : soutenir l'amélioration des soins de santé axés sur les individus et les populations
Committee/Comité: e-Health Innovations/Innovations en cybersanté
Title/Titre: Self-management and clinical decision support of multiple chronic conditions through the use of mobile health telemonitoring

---

Assessment/Évaluation:

Health Network (UHN), Toronto, ON. Patients (n=134) with at least two of the chronic

conditions will be recruited through the clinics and randomized.

The control group will receive usual care, and the intervention group will receive usual care plus the telemonitoring intervention. Both groups will be followed for 6 months. It is anticipated that 3 months will be required for trial preparation, 8 months starting January 2014 will be required for recruitment (based on 900 patients seen/year in the Heart Function Clinic, and over 600 patients seen/year in the CKD and COPD clinics, and approximately half of them with co-morbid conditions). About 1 month will be apportioned for data analysis and reporting

o the progress made to date.

The researchers have assembled an incredible team of experts, a boat-load of additional funding to support various aspects of this project, and have developed and tested one of the chronic condition modules.

2. **An assessment of the proposal, based on the evaluation criteria as presented in the funding opportunity details:** consider all factors and the strengths or weaknesses of the applications in relation to each criteria;

o this is a great research team, working in a great environment, on important topics.

While they are technically using information technology, the innovation in this project is not in the use of the technology itself (i.e., blood pressure and weight recording technologies are not new), but rather in how this will fit in with the patients lifestyle and the clinicians work flow. Unfortunately, they have not devoted many resources (other than a few interviews of patients and clinicians) to these aspects of the project. my reading of the literature on these types of projects seems to show that patients who use the technology seem to benefit from it, but only a small subset of patients are willing or able to make use of the devices. It seems to me that more research needs to be done to understand these issues or barriers.

**Review Type/Type d'évaluation:**	Committee Member 1/Membre de comité 1
**Name of Applicant/Nom du chercheur:**	SETO, Emily
**Application No./Numéro de demande:**	316619
**Agency/Agence:**	CIHR/IRSC
**Competition/Concours:**	2013-10-15 Catalyst Grant: e-Health Innovations: Supporting More Efficient Population and Individualized Healthcare/Subvention catalyseur : Innovations en cybersanté : soutenir l'amélioration des soins de santé axés sur les individus et les populations
**Committee/Comité:**	e-Health Innovations/Innovations en cybersanté
**Title/Titre:**	Self-management and clinical decision support of multiple chronic conditions through the use of mobile health telemonitoring

Assessment/Évaluation:

3. **Comments on the budget requested and a formal recommendation**, including clear and detailed reasons for any recommended budget cuts. (See Section 6 Budget and Term Determinations below).

Excellent, with much additional outside funding.

4. **If necessary, comments on issues the reviewer feels should be flagged**. For example, ethical issues, eligibility, compatibility with CIHR policies, etc. Such concerns, however, must not prevent a committee from evaluating

Review Type/Type d'évaluation: Committee Member 2/Membre de comité 2
Name of Applicant/Nom du chercheur: SETO, Emily
Application No./Numéro de demande: 316619
Agency/Agence: CIHR/IRSC
Competition/Concours: 2013-10-15 Catalyst Grant: e-Health Innovations: Supporting More Efficient Population and Individualized Healthcare/Subvention catalyseur : Innovations en cybersanté : soutenir l'amélioration des soins de santé axés sur les individus et les populations
Committee/Comité: e-Health Innovations/Innovations en cybersanté
Title/Titre: Self-management and clinical decision support of multiple chronic conditions through the use of mobile health telemonitoring

Assessment/Évaluation:

Self-Management and clinical decision support of multiple chronic conditions through the use of mobile health monitoring

PI: Seto Emily, Health Policy, Management & Evaluation, Uof Toronto (8 hr/week)
Co-Applicants:

- Ong Stephanie, Principal Knowledge User, Faculty of Medicine, UofT (4 hr /week)
- Cafazzo Josepth, Co-Applicant, Uof T (3 hrs /week)
- Casey Deborah, Co-Applicant, Faculty of Medicine, University Health Network (2 hr/week)
- Chapman Kenneth, Co-Applicant, Fac of Medicine, University Health Network (2 hrs /week)
- Logan Alexander, Co-Applicant, Mount Sinai Hospital (2 hrs /week)
- Miller Judith, Co-Applicant, Fac of Medicine, University Health Network (2 hrs /week)
- Ritvo Pual, Co-Applicant, York University (2 hrs/week)
- Ross heather, Co-Applicant, Fac of Medicine, University Health Network (2 hrs/week)
- Rotundi Michael, Collaborator (1 hr/week)

Synopsis of the Proposal

- Purpose: The intent is to design and deploy a user-centered mHealth tool that enables patients to monitor their MCCs (multiple chronic co-morbidities) using wireless medical peripheral devices, and automated questionnaires, and provides self-management instructions at the earliest signs of deterioration, prevention hospitalization. We also aim to reconcile confusion regarding conflicting self-care practices appropriate for different chronic conditionsPropose to develop a patient-centered care planning etool that will enhance the integration of care across care providers and sectors.
- Canadian business: Blackberry, QNX software systems, NexJ systems
- A small feasibility pilot will be conducted to validate the tool, revealing any issues in clinical workflow, telemonitoring system design, methods for patient recruitment etc. prior to the proposed RCTWhich
- Research question: What is the impact of a telemonitoring system for patients with MCCs, on self-management, clinical outcomes, and health service utilization?
- RCT will be conducted to determine the impact of Medly on healthcare costs and clinical outcomes.
- Patients (n=134) with at least 2 chronic conditions.
- CG usual care and IG usual care plus telemonitoring intervention

**Review Type/Type d'évaluation:**	Committee Member 2/Membre de comité 2
**Name of Applicant/Nom du chercheur:**	SETO, Emily
**Application No./Numéro de demande:**	316619
**Agency/Agence:**	CIHR/IRSC
**Competition/Concours:**	2013-10-15 Catalyst Grant: e-Health Innovations: Supporting More Efficient Population and Individualized Healthcare/Subvention catalyseur : Innovations en cybersanté : soutenir l'amélioration des soins de santé axés sur les individus et les populations
**Committee/Comité:**	e-Health Innovations/Innovations en cybersanté
**Title/Titre:**	Self-management and clinical decision support of multiple chronic conditions through the use of mobile health telemonitoring

Assessment/Évaluation:

- Outcomes:
  - Primary outcomes: Hospitalization rates, days in hospital, number of ED visits and clinic visits
  - Secondary outcomes: severity of patient conditions, QOL, self-care
- Participants in the IG will be interviewed individually to assess their experiences and perceptions regarding the use of Medly.
- Clinicians involved in the trial will also be interviews to determine their perceptions.
-
- <u>Assessment of Proposal – well-done</u>

- Will all clinicians involved in the trial be interviewed?

<u>Budget</u>

- Request CIHR 99,647$ - OK
- 1 technician, 2 other personnel
- 2 graduate students
- Cash and inkind contributions

Review Type/Type d'évaluation: SO Notes /Notes de l'agent scientifique
Name of Applicant/Nom du chercheur: SETO, Emily
Application No./Numéro de demande: 316619
Agency/Agence: CIHR/IRSC
Competition/Concours: 2013-10-15 Catalyst Grant: e-Health Innovations: Supporting More Efficient Population and Individualized Healthcare/Subvention catalyseur : Innovations en cybersanté : soutenir l'amélioration des soins de santé axés sur les individus et les populations
Committee/Comité: e-Health Innovations/Innovations en cybersanté
Title/Titre: Self-management and clinical decision support of multiple chronic conditions through the use of mobile health telemonitoring

---

Assessment/Évaluation:

SETO

This is a well-written proposal that addresses an important issue. The team should consider evaluating negative effects (such as anxiety produced by tele-monitoring). This is a strong team with substantial cash and in-kind contribution from other partners. It is unclear however whether the team has experience with RCT designs.
